# Gynoecium size and ovule number are interconnected traits that impact seed yield

**DOI:** 10.1093/jxb/eraa050

**Published:** 2020-02-18

**Authors:** Mara Cucinotta, Maurizio Di Marzo, Andrea Guazzotti, Stefan de Folter, Martin M Kater, Lucia Colombo

**Affiliations:** 1 Dipartimento di Bioscienze, Università degli Studi di Milano, Via Celoria, Milan, Italy; 2 Unidad de Genómica Avanzada (UGA-Langebio), Centro de Investigación y de Estudios Avanzados del Instituto Politecnico Nacional (CINVESTAV-IPN), Km. 9.6 Libramiento Norte, Carretera Irapuato-Leon, CP 36824 Irapuato, Gto., Mexico; 3 Trinity College Dublin, Ireland

**Keywords:** Gynoecium, hormones, organ boundary, ovule number, ovule primordia, pistil, seed yield

## Abstract

Angiosperms form the largest group of land plants and display an astonishing diversity of floral structures. The development of flowers greatly contributed to the evolutionary success of the angiosperms as they guarantee efficient reproduction with the help of either biotic or abiotic vectors. The female reproductive part of the flower is the gynoecium (also called pistil). Ovules arise from meristematic tissue within the gynoecium. Upon fertilization, these ovules develop into seeds while the gynoecium turns into a fruit. Gene regulatory networks involving transcription factors and hormonal communication regulate ovule primordium initiation, spacing on the placenta, and development. Ovule number and gynoecium size are usually correlated and several genetic factors that impact these traits have been identified. Understanding and fine-tuning the gene regulatory networks influencing ovule number and pistil length open up strategies for crop yield improvement, which is pivotal in light of a rapidly growing world population. In this review, we present an overview of the current knowledge of the genes and hormones involved in determining ovule number and gynoecium size. We propose a model for the gene regulatory network that guides the developmental processes that determine seed yield.

## Teaser

An overview of the gene and hormone regulatory network involved in determining ovule number and gynoecium size, two fundamental processes that affect seed yield.

## Introduction

Life on earth is affected by plants in varied ways. Of the estimated 400 000 extant plant species, approximately 94% are seed plants (Govaerts, 2001; Willis, 2017). This demonstrates that seed development and dispersion strategies greatly contributed to the success of this organismal group. The vast majority of seed plants are angiosperms and only a comparatively small number are gymnosperms. Both plant divisions produce ovules; however, only angiosperm species produce flowers, and as another selective advantage, each flower produces one or more gynoecia that protect and nourish the ovules. Following fertilization, the gynoecium (or pistil) generally develops into a fruit and ovules develop into seeds.

Depending on the species, the gynoecium consists of one or more carpels, which can be fused or unfused (Endress and Igersheim, 2000). The Arabidopsis gynoecium consists of two fused carpels (Smyth *et al.*, 1990; Alvarez-Buylla *et al.*, 2010). Along the margins where the carpels fuse, a meristematic tissue, termed the carpel margin meristem (CMM), is formed. The CMM gives rise to the placenta, ovules, septum, and transmitting tract (Reyes-Olalde *et al.*, 2013; Reyes-Olalde and de Folter, 2019). Inside an ovule the female gametophyte develops, comprising three antipodal cells, a central cell, two synergids, and an egg cell (Bencivenga *et al.*, 2011; Drews and Koltunow, 2011). Therefore, ovule development is a crucial process during the plant life cycle and has been studied in many species. In recent decades, many reviews on ovule development have been written, demonstrating its importance and the degree of active research in this area (e.g. Reiser and Fischer, 1993; Angenent and Colombo, 1996; Grossniklaus and Schneitz, 1998; Gasser *et al.*, 1998; Bowman *et al.*, 1999; Skinner *et al.*, 2004; Colombo *et al.*, 2008; Shi and Yang, 2011; Endress, 2011; Cucinotta *et al.*, 2014; Gasser and Skinner, 2019; Pinto *et al.*, 2019; Shirley *et al.*, 2019).

**Fig. 1.**
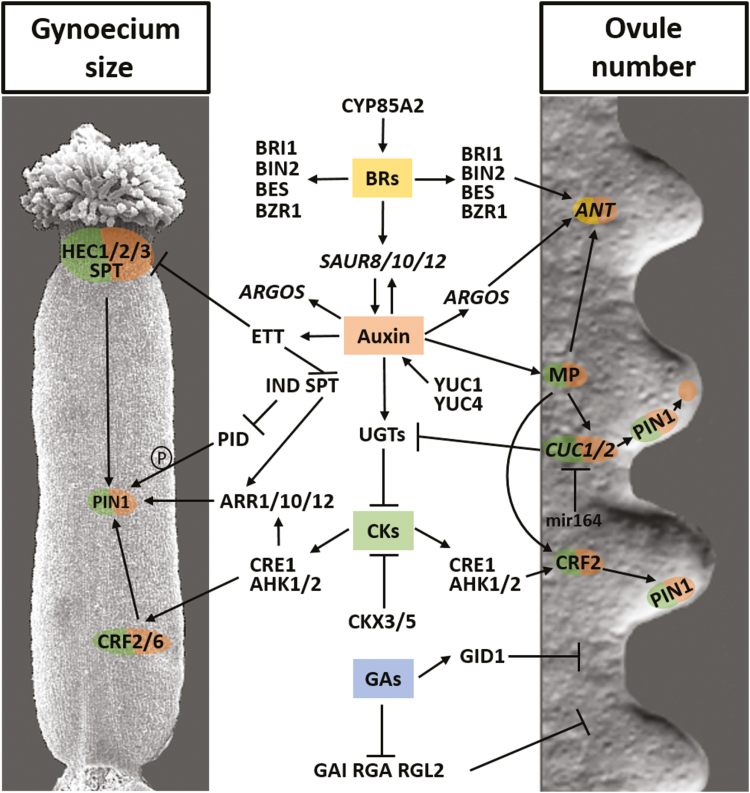
Proposed model for the regulation of pistil growth and ovule primordium initiation. A gynoecium of Arabidopsis is shown on the left while the image on the right depicts ovule primordia; in the centre, the interconnected gene network that regulates the two processes is shown. Auxin, through ETT, regulates gynoecium fusion and elongation by repressing *IND*, *HEC*s, and *SPT*, which in turn modulate polarization of the auxin efflux carrier PIN1 via repressing *PID*. CK positively regulates *PIN1* expression. In particular, the CK response mediated by CRFs and ARRs is directly required for pistil elongation and indirectly affects ovule primordium initiation. *CRF2* regulation by MP further integrates the auxin–CK crosstalk. Moreover, MP directly regulates *CUC1* and *CUC2* expression. In turn, CUCs control *PIN1* expression and PIN1 protein localization, which is required for correct ovule primordium development. CUCs positively influence the CK pathway by transcriptionally repressing the CK-inactivating glycosyltransferase enzymes (UGTs). *ANT*, whose expression is controlled by auxin and BRs, is required for cell division in ovule primordia. *ANT* is also regulated by auxin via MP and ARGOS. BR signalling also positively affects pistil elongation. GA has a negative effect on ovule number, but its connection with other hormones remains to be addressed.

To complement existing literature, this review focuses on recent discoveries in ovule development and gynoecium size determination. An overview is provided of the genes and hormonal communication involved in the developmental programs (Fig. 1; Table 1). Understanding the regulatory networks that determine ovule number and gynoecium size is important as they hugely impact seed yield, and fine-tuning them appears to be a particularly promising strategy for enhancing crop yields.

**Table 1.** Genes involved in determining gynoecium size and/or ovule number

Gene name	Family or protein type	Gynoecium size	Ovule number	Reference
*ANT*	AP2/EREBP transcription factor	*ant-9* ↓ *ant-4* ↓ *35S::ANT* ↑	*ant-1* ↓ *ant-3* ↓ *ant-4* ↓ *ant-9* ↓	Elliott *et al.* (1996), Liu *et al.* (2000), Azhakanandam *et al.* (2008), Krizek (2009), Wynn *et al.* (2014)
*ARGOS*	ARGOS protein	*35S::ARGOS* ↑		Hu *et al.* (2003)
*CRC*	YABBY transcription factor	*crc-1* ↓		Gross *et al.* (2018)
*SPT*	bHLH transcription factor	*spt-2* ↓	*spt-2* ↓	Heisler *et al.* (2001), Alvarez and Smyth (2002), Nahar *et al.* (2012)
*ETT* (*ARF3*)	ARF transcription factor	*ett-1* ↓ *ett-2* ↓		Sessions *et al.* (1997), Nemhauser *et al.* (2000)
*HEC1*, *HEC2*, *HEC3*	bHLH transcription factor	*hec1 hec2 hec3* ↓		Gremski *et al.* (2007)
*ARR1*, *ARR10*, *ARR12*	Type-B ARR transcription factor	*arr1 arr10 arr12* ↓	*arr1 arr10 arr12* ↓	Reyes-Olalde *et al.* (2017)
*CRF2*, *CRF3*, *CRF6*	ERF transcription factor	*crf2 crf3 crf6* ↓	*crf2 crf3 crf6* ↓	Cucinotta *et al.* (2016)
*PIN1*	PIN auxin efflux carrier	*pin1* ↓	*pin1* ↓ *pin1-5* ↓	Okada *et al.* (1991), Bencivenga *et al.* (2012), Cucinotta *et al.* (2016)
*CKX3*, *CKX5*	CKX cytokinin oxidase/dehydrogenase protein	*ckx3 ckx5* ↑	*ckx3 ckx5* ↑	Bartrina *et al.* (2011)
*UGT85A3*, *UGT73C1*	UDP-glucosyl transferase	*35S::UGT85A3* ↓ *35S::UGT73C1* ↓	*35S::UGT85A3* ↓ *35S::UGT73C1* ↓	Cucinotta *et al.* (2018)
*SAUR8*, *SAUR10*, *SAUR12*	SAUR-like auxin-responsive protein family	*35S::SAUR8* ↑ *35S::SAUR10* ↑ *35S::SAUR12* ↑		van Mourik *et al.* (2017)
*BZR1*	Brassinosteroid signalling regulatory protein	*bzr1-1D* ↑	*bzr1-1D* ↑	Huang *et al.* (2013)
*BIN2*	ATSK (shaggy-like kinase) family	*bin2* ↓	*bin2* ↓	Huang *et al.* (2013)
*DET2*	3-Oxo-5-α-steroid 4-dehydrogenase protein	*det2* ↓	*det2* ↓	Huang *et al.* (2013)
*BRI1*	Leucine-rich receptor-like protein kinase protein	*bri1-5* ↓	*bri1-5* ↓	Huang *et al.* (2013)
*CYP85A2*	Cytochrome p450 enzyme		*cyp85a2-1* ↓ *cyp85a2-2* ↓	Nole-Wilson *et al.* (2010b)
*SEU*	Transcriptional adaptor	*seu-1* ↓	*seu-1* ↓	Nole-Wilson *et al.* (2010b)
*CTR1*	RAF homologue of serine/threonine kinase	*ctr1-1* ↓		Carbonell-Bejerano *et al.* (2011)
*REV*	Homeobox-leucine zipper protein		*ant rev* ↓	Nole-Wilson *et al.* (2010a)
*L–UG*	WD40/YVTN repeat-like-containing domain transcription factor		*lug-1* ↓ *lug-3* ↓	Azhakanandam *et al.* (2008)
*PAN*	bZIP transcription factor	*ant pan* ↓ *seu pan* ↓	*ant pan* ↓ *seu pan* ↓	Wynn *et al.* (2014)
*HLL*	Ribosomal protein L14p/L23e	*hll* ↓	*hll* ↓	Schneitz *et al.* (1998), Skinner *et al.* (2001)
*SIN2*	P-loop containing nucleoside triphosphate hydrolase superfamily protein	*sin-2* ↓	*sin-2* ↓	Broadhvest *et al.* (2000)
*YUC1*, *YUC4*	Flavin-binding monooxygenase protein		*yuc1 yuc4* ↓	Cheng *et al.* (2006)
*AHK2*, *AHK3*, *CRE1*	Histidine kinase		*cre1-12 ahk2-2 ahk3-3* ↓	Bencivenga *et al.* (2012)
*CUC1*, *CUC2*	NAC transcription factor		*cuc1 cuc2* ↓ *pSTK::CUC1/RNAi cuc2-1* ↓	Galbiati *et al.* (2013)
*MIR164A*	microRNA		*35S::MIR164A* ↓	Gonçalves *et al.* (2015)
*GAI*, *RGA*, *RGL2*	GRAS transcription factor	*gaiT6 rgaT2 rgl2-1* ↓	*gaiT6 rgaT2 rgl2-1* ↓	Gomez *et al.* (2018)
*GID1A*, *GID1B*	α/β-Hydrolase superfamily protein		*gid1ab* ↑	Gomez *et al.* (2018)
*REM22*	B3 protein transcription factor		*rem22-1* ↑	Gomez *et al.* (2018)
*UNE16*	Homeodomain-like superfamily protein		*une16-1* ↓	Gomez *et al.* (2018)
*NERD1*	GW repeat- and PHD-finger-containing protein NERD		*nerd1-2* ↓ *nerd1-4* ↓	Yuan and Kessler (2019)
*ONA2*	Unknown protein		*ona2* ↓	Yuan and Kessler (2019)
*ASHH2*	Histone-lysine *N*-methyltransferase		*ashh2* ↓	Grini *et al.* (2009)

Up- and down-pointing arrows represent how the mutant phenotype impacts either gynoecium size or ovule number.

## Placenta development and ovule primordium initiation in Arabidopsis

Periclinal cell divisions within the sub-epidermal tissue of the placenta initiate ovule primordium development at stage 9 of flower development (Roeder and Yanofsky, 2006). Subsequently, three layers of primordium cells form a finger-like structure during stage 10, which then differentiates into three regions along the proximal–distal axis: the funiculus, the chalaza, and the nucellus (Schneitz *et al.*, 1995). These three regions undergo distinct but interdependent developmental processes. The nucellus is the site of megasporogenesis, where the megaspore mother cell differentiates and locates to the upmost, central, and subepidermal position of the digit-shaped ovule primordium (reviewed in Pinto *et al.*, 2019). The chalaza is the region from which the inner and outer integuments develop, and these finally envelop and protect the embryonic sac. The funiculus remains attached to the gynoecium via the placental tissue and this connection is required for the transport of nutrients to the ovule (Fig. 1). For this reason, the placental tissue is fundamental for ovule primordia formation, and for determining their number and maintenance.

In Arabidopsis, placental tissue differentiates from the CMM, which is the central ridge of cells that fuse and give rise to the septum. Placental tissue differentiates along the length of the septum adjacent to the lateral walls (Alvarez and Smyth, 2002; Nole-Wilson *et al.*, 2010a; Reyes-Olalde *et al.*, 2013). Communication between transcription factors and hormones is essential to maintain the meristematic activity of the placenta, to determine the sites of ovule initiation and ovule identity, and to establish the distance between two adjacent ovules (Cucinotta *et al.*, 2014). Several genes that are important for placenta development have been described in the literature (reviewed by Cucinotta *et al.*, 2014; Reyes-Olalde and de Folter, 2019), including *AINTEGUMENTA* (*ANT*), *CUP-SHAPED COTYLEDON 1* (*CUC1*) and *CUC2*, *LEUNIG* (*LUG*), *MONOPTEROS* (*MP*), and *PERIANTHIA* (*PAN*) (Fig. 1; Table 1).

*AINTEGUMENTA* encodes an AP2 transcription factor (Klucher *et al.*, 1996) and positively regulates organ size via determining cell number and meristematic competence. *Ant* mutants have fewer and smaller floral organs than the wild-type. In particular, the *ant-9* mutant is characterized by unfused carpels at the tip of the pistil (Elliott *et al.*, 1996), whereas in *ant-4*, the size of floral organs is reduced (Krizek, 2009). In contrast to these mutant phenotypes, Arabidopsis plants that overexpress *ANT* possess larger floral organs than the wild-type (Mizukami and Fischer, 2000). Expression of *ANT* is controlled by *AUXIN-REGULATED GENE INVOLVED IN ORGAN SIZE* (*ARGOS*), an auxin-inducible gene (Hu *et al.*, 2003). When *ARGOS* is overexpressed, floral organs become enlarged, resulting in longer siliques than those of wild-type (Hu *et al.*, 2003). This was one of the first pieces of evidence that implicated a key role for auxin in pistil development.

*ANT* expression initiates in the placenta and is maintained throughout all stages of ovule development, in particular in the chalaza region and in the integuments. The reduced ovule number phenotype of the *ant* mutant is exacerbated when it is combined with other mutations that affect CMM and placenta development, such as *revoluta* (*rev*), suggesting that the activity of the *REV* gene, which encodes a class III homeodomain leucine zipper transcription factor, is also required for placenta formation (Nole-Wilson *et al.*, 2010a). ANT interacts with the transcriptional repressor SEUSS (SEU) and simultaneous loss of both protein activities severely affects placenta development and leads to a complete loss of ovule formation. When a weaker *ant-3* allele was combined with *seu-3*, placenta development was maintained but the number of ovules that initiated was reduced to approximately half of that observed in Col-0 wild-type plants (Azhakanandam *et al.*, 2008). Another transcriptional co-regulator involved in gynoecium patterning is LEUNIG (LUG). Strong *lug-1* and intermediate *lug-3* alleles show a failure in ridge fusion and a reduction in the amount of placental tissue, with a consequent decrease in the number of ovules formed (Liu *et al.*, 2000). The combination of *lug* and *ant* mutations results in gynoecia that are unable to develop ovules (Liu *et al.*, 2000). The loss of ovules in the *ant* and *seu* backgrounds is strongly enhanced by mutations in the *PERIANTHIA* (*PAN*) gene, which encodes a bZIP transcription factor that is expressed in the gynoecium medial ridge, placenta, and ovules, where it promotes ovule formation (Wynn *et al.*, 2014).

Similar to *ANT*, factors important for integument growth often affect ovule primordium formation. Two examples are *HUELLENLOS* (*HLL*) and *SHORT INTEGUMENTS 2* (*SIN2*). *HLL* encodes a mitochondrial ribosomal protein and its mutation is associated with smaller gynoecia and a 10% reduction in the number of ovules (Schneitz *et al.*, 1998; Skinner *et al.*, 2001). Shorter gynoecia that bear fewer ovules are also observed in the *sin2* mutant; however, more interestingly, the absence of *SIN2* function leads to an abnormal distribution of ovules along the placenta (Broadhvest *et al.*, 2000), in which the distance between ovules is greater than in the wild-type; thus, a reduction in ovule number is caused by a reduction in gynoecium size and by the reduced ability of the placental tissue to initiate ovule primordia. *SIN2* encodes a mitochondrial DAR GTPase and, similar to HLL, is hypothesized to function in mitochondrial ribosome assembly (Hill *et al.*, 2006). Notably, these two ribosomal proteins, which are targeted to the mitochondria, are necessary for ovule primordium formation, and it has been suggested that impaired mitochondrial function might cause cell-cycle arrest in the placenta and subsequently in the ovule integuments (Broadhvest *et al.* 2000).

## Complex hormonal communication promotes ovule initiation and determines pistil size

Plant organogenesis requires cells to proliferate, grow, and differentiate in a coordinated way. The intercellular communication required during organ initiation is mediated by different phytohormones (Davies, 2004; Vanstraelen and Benková, 2012; Schaller *et al.*, 2015; Marsch-Martínez and de Folter, 2016). As will be discussed in this review, auxins, cytokinins (CKs), gibberellins (GAs), and brassinosteroids (BRs) all play fundamental roles in ovule primordium formation (Fig. 1).

In most auxin-related mutants, defects in gynoecium formation lead to the reduction or absence of placental tissue and the corresponding absence of ovules (reviewed in Balanzá *et al.*, 2006; Larsson *et al.*, 2013; Cucinotta *et al.*, 2014). This phenotype is common to all mutants in which auxin synthesis or transport pathways are compromised, such as *yucca1 yucca4* (*yuc1 yuc4*) (Cheng *et al.*, 2006) and *pin1-1* (Okada *et al.*, 1991) or is similar to that following treatment with the polar auxin transport inhibitor 1-naphthyl phthalamic acid (NPA) (Nemhauser *et al.*, 2000).

Polar auxin transport is mediated by the PINFORMED1 (PIN1) efflux transporter and is required to create a zone with an auxin concentration maximum in the placenta, where the founder cells of the ovule primordia will be specified (Benková *et al.*, 2003; Ceccato *et al.*, 2013; Galbiati *et al.*, 2013). Subsequently, the orientation of PIN1 within the membrane relocalizes and redirects auxin flow, establishing a gradient with a maximum at the apices of the formed primordia. In developing organs, auxin distribution can be monitored *in vivo* by imaging a synthetic auxin-inducible promoter, *DR5*. In plants that express green fluorescent protein (GFP) from the *DR5* promoter, green fluorescence is detected at the apices of the ovule primordia, consistent with PIN1-mediated auxin flow directed to the apex (Benková *et al.*, 2003; Galbiati *et al.*, 2013). The weak *pin1-5* mutant allele can produce some flowers in which the pistils have slightly reduced valves, which on average contain only nine ovules (Bennett *et al.*, 1995; Sohlberg *et al.*, 2006; Bencivenga *et al.*, 2012).

CKs occupy a central role in the regulation of cell division and cell differentiation. They are positive regulators of ovule formation, as demonstrated by the phenotype of mutants in which CK pathways are altered. In the *ckx3 ckx5* double mutant, the degradation of CKs is compromised and the consequent increase in the levels of these hormones leads to an increased activity of the reproductive meristem (Bartrina *et al.*, 2011). Moreover, the longer than normal gynoecia of *ckx3 ckx5* double mutants contain about twice as many ovules as those of the wild-type, indicating an increase in the meristematic capacity of placental tissue (Bartrina *et al.*, 2011). By contrast, reduced ovule formation is observed in mutants in which the synthesis or perception of CKs is compromised. Plants that carry mutations in genes that encode all three CK receptors, *cytokinin response 1* (*cre1-12*), *histidine kinase2* (*ahk2-2*), and *ahk3*, develop five ovules per pistil on average, in addition to showing pleiotropic growth defects (Higuchi *et al.*, 2004; Bencivenga *et al.*, 2012). The AHK2 and AHK3 receptors are expressed throughout ovule development, from the early stages until maturity, whereas *CRE1*/*AHK4* is expressed in the chalaza region and subsequently in the integuments, suggesting that AHK2 and AHK3 preferentially contribute to ovule primordium formation (Bencivenga *et al.*, 2012). The ovule and gynoecium phenotype of the *cre1-12 ahk2-2 ahk3-3* triple mutant resembles that of the weak *pin1-5* mutant allele (Bencivenga *et al.*, 2012). This similarity is due to the downregulation of *PIN1* expression in the triple mutant, suggesting that during the early stages of ovule development, CK activates *PIN1* expression. Bencivenga *et al.* (2012) showed that treating inflorescences with the synthetic CK 6-benzylaminopurine (BAP) increases *PIN1* expression in the gynoecium. Strikingly, treatment with BAP causes the formation of on average 20 additional ovule primordia in each gynoecium, which are positioned between the existing primordia formed before the treatment. This suggests that placental tissue at the boundaries between ovules maintains meristematic competence. During root development, CK affects auxin polar transport via PIN1 both at the transcriptional and post-transcriptional levels. In contrast to the situation in the gynoecium, CK negatively regulates the expression of *PIN1* in the root and controls the endorecycling of PIN1 from the membrane to direct it to vacuoles for lytic degradation (Ruzicka *et al.*, 2009; Marhavý *et al.*, 2011). In roots, CYTOKININ RESPONSE FACTORS (CRFs), especially CRF2, CRF3, and CRF6, transcriptionally regulate *PIN1* by binding to its promoter at the *cis*-regulatory *PIN CYTOKININ RESPONSE ELEMENT* (*PCRE*) (Šimášková *et al.*, 2015) and modulate its expression in response to CK. Similarly, CRFs also mediate *PIN1* expression in ovules in response to CK (Cucinotta *et al.*, 2016). Indeed, *PIN1* expression is reduced in the *crf2 crf3 crf6* (*crf2/3/6*) triple mutant and cannot be increased by CK treatment. The placenta in *crf2/3/6* is also shorter, but this is not sufficient to explain the 30% decrease in ovule number as ovule density is lower in *crf2/3/6* than in the wild-type (Cucinotta *et al.*, 2016). Because *PIN1* expression in *crf2/3/6* was unresponsive to CK application, the mutant was significantly less sensitive to CK treatment than the wild-type with regard to an increase in ovule number and pistil length. Auxin also regulates *CRF2*, which is a direct target of the auxin response factor (ARF) AUXIN RESPONSE FACTOR 5/MONOPTEROS (ARF5/MP) (Schlereth *et al.*, 2010), highlighting another convergence point between auxin and CK.

Another ARF family member that is required for appropriate apical–basal gynoecium patterning is ARF3/ETTIN (ETT). The *ett* mutant is characterized by a shorter ovary with an elongated style and gynophore (Sessions *et al.*, 1997). A similar gynoecium phenotype resulted from treatment with the auxin transport inhibitor (NPA), suggesting that ETT plays a key role in auxin signalling along the apical–basal gynoecium axis (Nemhauser *et al.*, 2000). Moreover, ETT restricts the expression domain of *SPATULA* (*SPT*), which encodes a basic helix–loop–helix (bHLH) transcription factor (Heisler *et al.*, 2001). Mutations in *SPT* causes a split-carpel phenotype in the apical part of the gynoecium, leading to a slight reduction in ovule number (Alvarez and Smyth, 1999; Nahar *et al.*, 2012). SPT dimerizes with another bHLH transcription factor, INDEHISCHENT (IND), to repress the expression of *PINOID* (Girin *et al.*, 2011), which encodes a serine/threonine kinase that regulates PIN1 polarization via phosphorylation (Friml *et al.*, 2004). The repression of *PID* by SPT and IND allows the formation of a radially symmetric auxin ring in the upper part of the gynoecium that is required for correct style and stigma development (Moubayidin and Østergaard, 2014).

Furthermore, SPT interacts with the three closely related bHLH transcription factors, HECATE1 (HEC1), HEC2, and HEC3 (Gremski *et al.*, 2007), and similar to *ett*, *hec-1 hec-2 hec-3* triple mutants possess an elongated style and shorter ovaries. The HEC proteins and SPT promote auxin transport in concert by activating *PIN1* and *PIN3* expression (Schuster *et al.*, 2015) and also transcriptionally activate the type-A ARABIDOPSIS RESPONSE REGULATORS (ARR-As), which are negative regulators of CK signalling (Schuster *et al.*, 2015). Via this dual action on auxin transport and CK response, HECs and SPT regulate wild-type gynoecium fusion at the apex, and style and stigma development. Furthermore, SPT alone in the medial domain activates the type-B ARRs, especially ARR1, which are positive regulators of CK signalling. The *arr1 arr10 arr12* triple mutant possesses a shorter gynoecium and significantly fewer ovules than the wild-type (Reyes-Olalde *et al.*, 2017).

In addition to auxin localization, correct auxin signalling is also required for wild-type gynoecium development, as confirmed by a recent study on members of the Small Auxin-Upregulated RNA (SAUR) family, which were initially identified as short transcripts that were rapidly upregulated in response to auxin (McClure and Guilfoyle, 1987). When *SAUR8*, *SAUR10*, and *SAUR12* are ectopically overexpressed in Arabidopsis, the gynoecium and resulting siliques are longer than in wild-type, suggesting that auxin positively regulates gynoecium length and, probably indirectly, silique length (van Mourik *et al.*, 2017). Notably, *SAUR* gene expression increased 100-fold following combined auxin and BR treatment (van Mourik *et al.*, 2017). BRs are clearly involved in pistil growth and ovule number specification; gynoecia of the enhanced BR-signalling mutant *brassinazole-resistant 1-1D* (*bzr1-1D*) not only contained more ovules than wild-type but they were also longer. By contrast, BR-deficient mutants such as *de-etiolated 2* (*det-2*), *brassinosteroid insensitive 1* (*bri1-5*) and *brassinosteroid-insensitive 2* (*bin2-1*) developed shorter pistils with fewer ovules (Huang *et al.*, 2013).

The involvement of BRs in gynoecium and ovule development was also confirmed by Nole-Wilson *et al.* (2010b), who observed that a reduction in the expression of *CYP85A2*, which encodes an enzyme involved in the final step of brassinolide biosynthesis (Nomura *et al.*, 2005), enhances the *seuss* mutant phenotypic disruptions in ovules and gynoecia (Nole-Wilson *et al.*, 2010b).

## CUP-SHAPED COTYLEDON 1 and 2 function synergistically with auxin and cytokinins

During ovule primordium formation, CK homeostasis requires two NAC-domain transcription factors, CUP-SHAPED COTYLEDON 1 (CUC1) and CUC2. These are expressed in lateral organ boundaries and function redundantly during organ boundary determination. *CUC1* and *CUC2* are expressed in the septum and placenta, and following the emergence of ovule primordia, *CUC2* expression is restricted to the borders between the ovules (Ishida *et al.*, 2000; Galbiati *et al.*, 2013; Gonçalves *et al.*, 2015). The *CUC1* and *CUC2* genes are both post-transcriptionally regulated by *miR164* microRNAs (Laufs *et al.*, 2004; Mallory *et al.*, 2004). Gynoecia of the *in vitro* regenerated *cuc1 cuc2* mutant as well as of *cuc2-1 pSTK::CUC1_RNAi* plants have reduced ovule numbers. The c*uc1 cuc2* double mutant has on average fewer than 10 ovules per pistil (Ishida *et al.*, 2000), whereas *cuc2-1 pSTK::CUC1_RNAi* plants, in which *CUC1* was specifically silenced in the placenta and ovules, showed a 20% reduction in ovule number, but gynoecium length was not affected. In pistils of these plants, ovules were more widely spaced when compared with the wild-type (Galbiati *et al.*, 2013). This result was confirmed by silencing *CUC1* and *CUC2* by overexpressing *MIR164A*, which strongly reduced ovule number, indicating a major contribution of *CUC1* and *CUC2* to ovule initiation (Gonçalves *et al.*, 2015). The analysis of PIN1–GFP expression in *cuc2-1 pSTK::CUC1_RNAi* plants revealed that CUC1 and CUC2 redundantly promote *PIN1* expression and PIN1 membrane localization in ovules. Treatment with BAP increased *PIN1* expression and complemented the reduced ovule number phenotype of *cuc2-1 pSTK::CUC1_RNAi* plants (Galbiati *et al.*, 2013). Therefore, CKs act downstream from or in parallel with CUC1 and CUC2 to induce the expression of *PIN1*. Recently, it has been demonstrated that CUC1 and CUC2 induce CK responses *in vivo* and function upstream of CK by transcriptionally repressing *UGT73C1* and *UGT85A3*, which encode two enzymes involved in CK inactivation (Cucinotta *et al.*, 2018). Consistent with this result, the concentration of inactive CK glucosides was higher in *cuc2-1 pSTK::CUC1_RNAi* inflorescences than in wild-type plants.

The expression of *CUC1* and *CUC2* is also linked with auxin signalling: their expression pattern coincides with that of the auxin response factor ARF5/MP (see above) and both genes are downregulated in pistils of the weak *mp-S319* mutant allele (Galbiati *et al.*, 2013). During the early stages of placenta development and ovule formation, ARF5/MP directly transcriptionally activates *CUC1* and *CUC2*, but also *ANT*. The observation that BAP treatment did not complement the ovule number phenotype of *ant-4* suggests that ANT functions independently of *CUC1* and *CUC2*. This is further supported by the additive effects on the reduction in ovule number observed in *ant-4 cuc2-1 pSTK:CUC1_RNAi* plants (Galbiati *et al.*, 2013). Together these data suggest that ANT promotes cell proliferation, whereas CUC1 and CUC2 regulate CK homeostasis and auxin transport. Although CUC3 shares high similarity with CUC1 and CUC2, the *cuc3* mutant was not affected in ovule initiation and number, but together with CUC2, CUC3 promotes ovule separation; this is reflected by the *cuc2 cuc3* double mutant, which produces seeds that result from the fusion of two ovules (Gonçalves *et al.*, 2015). These results suggest that specific *CUC* genes independently promote ovule initiation and ovule separation.

Lee *et al.* (2009) identified LATERAL ORGAN FUSION 1 (LOF1) to be involved in lateral organ separation and to functionally overlap with CUC2 and CUC3. The *LOF1* gene is expressed at the base of ovule primordia and its overexpression results in a wrinkled pistil with an enlarged replum, an amorphous septum and an irregular ovule distribution (Gomez *et al.*, 2011).

## The role of gibberellins in ovule primordium formation

GAs are involved in key developmental processes throughout the plant life cycle, from seed germination in particular, to flowering time (reviewed in Hedden and Sponsel, 2015; Rizza and Jones, 2019), but their involvement in ovule initiation has only recently been demonstrated. Gomez and colleagues (2018) showed that DELLA proteins, which belong to a subfamily of the plant-specific GRAS family of transcriptional regulators that repress GA signalling, positively regulate ovule number in Arabidopsis. In addition to DELLA proteins, the GA signalling core includes the GA receptor GID1. When GID1 binds bioactive GA, the GA–GID1–DELLA complex is formed and triggers the polyubiquitination and degradation of DELLA proteins. The *della* triple mutant *gaiT6 rgaT2 rgl2-1* produces fewer ovules than wild-type (Gomez *et al.*, 2018). By contrast, the gain-of-function DELLA mutant *gai-1*, which cannot be degraded upon GA sensing, produced more ovules. Consistent with this observation, the double *gid1a gid1b* mutant, which cannot perceive GA, forms more ovules than the wild-type, demonstrating a negative correlation between GAs and ovule number (Gomez *et al.*, 2018). The *GAI*, *RGA*, *RGL2*, *GID1a*, and *GID1b* genes are expressed in placental tissue and outgrowing ovules. The reduction in ovule number was more dramatic in the *gaiT6 rgaT2 rgl2-1* triple mutant than that in ovary length, resulting in a lower ovule density, whereas the dominant *gai-1* mutant has an increased ovule/placenta ratio, suggesting that GAs predominantly affect ovule initiation and not placenta elongation.

Other evidence to demonstrate that DELLA proteins promote ovule formation derives from an experiment in which the expression of the stable mutant protein *rgaΔ17* under the control of the *ANT* promoter in the placenta resulted in the formation of 20% more ovules than in control lines (Gomez *et al.*, 2018). This effect of GAs on the number of developing ovules was not correlated with auxin signalling or transport, and neither PIN1 localization nor *DR5* expression was affected by GA treatment or DELLA activity (Gomez *et al.*, 2018).

Confirmation of a positive role for *RGL2* in determining ovule number came from the analysis of transgenic lines in which RGL2-dependent GA signalling was blocked by the expression of a dominant version of RGL2 (*pRGL2:rgl2Δ17*) (Gómez *et al.*, 2019). Pistils of *pRGL2:rgl2Δ17* plants contained 10% more ovules than those of the wild-type, whereas pistil length did not differ, indicating that the main function of rgl2Δ17 is to positively promote ovule primordium formation but not placenta elongation (Gómez *et al.*, 2019). Furthermore, Gomez *et al.* (2018) identified *REPRODUCTIVE MERISTEM 22* (*REM22*) and *UNFERTLIZED EMBRYO SAC 16* (*UNE16*) via transcriptomic analysis to be DELLA targets that are positive regulators of ovule initiation. REM22 is a B3 family transcription factor that is expressed in the placenta (Mantegazza *et al.*, 2014) and increased *REM22* expression in the *rem22-1* enhancer allele significantly increases ovule number. UNE16 is a transcription factor involved in embryo sac development and the knockdown allele *une16-1* produces fewer ovules. Because *UNE16* expression is regulated by BRs (Pagnussat *et al.*, 2005; Sun *et al.*, 2010), it represents a potential nexus for crosstalk between GAs and BRs in ovule initiation. The establishment of GA as an important additional component of the ovule regulatory network has introduced an additional layer of complexity to the current model for ovule initiation and it remains to be established how GAs integrate into this model. GAs might function antagonistically to CKs and BRs, which in contrast to GAs, positively regulate pistil size and ovule number.

Finally, the *ctr1-1* constitutive ethylene-responsive mutant possesses a shorter gynoecium at anthesis compared with wild-type and a delay in the response to GA_3_ treatment that induces gynoecium senescence, suggesting that ethylene affects gynoecium size, probably by interactions with GA pathways (Carbonell-Bejerano *et al.*, 2011).

In conclusion, there is ample evidence for complex interactions between different hormonal pathways that together determine ovule number and pistil size.

## Ovule number: the ecotype matters

It has been known for 20 years that the number of ovules varies hugely among different Arabidopsis ecotypes (diploid accessions) (Alonso-Blanco *et al.*, 1999): for example, the Landsberg *erecta* accession produces 20% more ovules than the Cape Verde Islands (Cvi) accession. Recently, 189 Arabidopsis accessions from the Arabidopsis Biological Resource Center were analysed for differences in ovule number and they display a remarkable degree of variation, ranging from 39 to 82 ovules per pistil (Yuan and Kessler, 2019). The commonly used reference accession Col-0 lies in the middle of the range, with a mean ovule number of 63, which is strongly dependent on experimental growth conditions. Ovule number, in contrast to, for instance, flowering time, does not correlate with geographical origin (Stinchcombe *et al.*, 2004; Yuan and Kessler, 2019). By conducting a genome-wide association study on these 189 accessions, two loci associated with ovule number were identified (Yuan and Kessler, 2019): *NEW ENHANCER OF ROOT DWARFISM* (*NERD1*) and *OVULE NUMBER ASSOCIATED 2* (*ONA2*). Mutation of *NERD1* or *ONA2* leads to a significant reduction in ovule number, with a stronger phenotype in the *nerd1-2* and *nerd1-4* alleles. *ONA2* encodes a protein of unknown function and was not further analysed. In addition to a reduction in ovule number, *nerd* mutants display additional severe male and female fertility defects. *NERD1* encodes an integral membrane protein mainly localized to the Golgi. Notably, *NERD1* expression is lower in Altai-5 and Kas-2 accessions, which have low ovule numbers (Yuan and Kessler, 2019), but high *NERD1* expression in Altai-5 leads to a significant increase in ovule number. However, overexpression of NERD1 in Col-0 plants did not affect ovule number, indicating that NERD1 function in determining ovule number is background-dependent (Yuan and Kessler, 2019).

Considerable genetic variation in ovule number was also described for F_1_ triploids of different Arabidopsis genotypes by Duszynska *et al.* (2013), who observed differences in ovule number between genetically identical F_1_-hybrid offspring, after crossing parental genome excess lines (2m:1p with 1m:2p). These effects can only be explained by epigenetic mechanisms that affect genes controlling ovule number, for example DNA or histone methylation. The analysis of null alleles of *ASH1 HOMOLOG 2* (ASH2), which show a remarkable 80% reduction in ovule number, provided a clear example of the involvement of histone methylation in determining ovule number (Grini *et al.*, 2009). The transcriptional state of the *ASH2* locus remains active during development via H3K36 trimethylation (Xu *et al.*, 2008). It will be highly relevant to study the effect of epigenetic modifications induced by biotic and abiotic stresses in determining ovule number. Epigenetic responses to stress are fundamental to create the plasticity required for plant survival, especially considering that plants are sessile organisms. These epigenetic changes can be temporally transmitted, even in the absence of the original stress (Iglesias and Cerdán, 2016). Furthermore, variation in ovule number in response to fluctuations in environmental conditions, such as temperature, can be used to understand the plasticity and inheritability of (epigenetic) adaptation and response to temperature stress. Variation in ovule number under stress conditions is, of course, also highly relevant from an ecological, environmental, and evolutional perspective.

## Ovule number decreases with ageing

Ovule number varies throughout inflorescence development: early flowers developing on the main inflorescence (from the fifth to the twenty-fifth flower) of Arabidopsis L*er* plants produced a relatively invariable number of ovules, whereas flowers that developed later had pistils with fewer ovules (Gomez *et al.*, 2018; Yuan and Kessler, 2019). Loss- and gain-of-function mutants of *DELLA* genes showed an increase in ovule number in early- and late-arising flowers (Gomez *et al.*, 2018). To minimize age-related variation in their genome-wide association studies, Yuan and Kessler (2019) only counted ovules in flowers 6–10 from the main inflorescence.

It has been reported for other plant species that flower position as well as size influences ovule number per flower. For example, in pomegranate, the number of ovules per flower was significantly influenced by flower size, with more ovules being produced in larger flowers (Wetzstein *et al.*, 2013).

Overall, when studying changes in ovule numbers it is important to be aware of the possible variation in the different flowers of the plant. Therefore, large numbers will have to be analysed using thorough statistical analyses, especially for genotypes that show only relatively minor changes.

## A ‘gold mine’ for seed yield improvement within the Brassicaceae

Improving seed yield via the genetic manipulation of crops has historically been a central goal in agricultural research. The enormous body of data, which has been generated and shared by the scientific community over the past decades, represents a true ‘gold mine’ for translational and applied research. The determination of pistil size and ovule number may be considered one of the most straightforward traits that can be enhanced to improve overall seed yield in species characterized by multi-ovulate ovaries and the increasing amount of literature on this topic evidences an active and prolific research field. Although some questions concerning the networks controlling seed number and pistil size remain open, comprehensive knowledge of the phytohormone interactions involved in these pathways is already available and applicable (Cucinotta *et al.*, 2014; Zúñiga-Mayo *et al.*, 2019; Reyes-Olalde and de Folter, 2019).

Understanding these developmental processes in Arabidopsis can inform promising strategies for knowledge transfer to closely related and agronomically important crops. Rapeseed (*Brassica napus*), another Brassicaceae species, is an important breeding target, since it is a crop widely cultivated in Europe, Asia, Canada, and Australia. It is characterized by an oil-rich seed and its processing provides both rapeseed oil (used as edible vegetable oil or as biodiesel) and a by-product mostly used as cattle fodder (Snowdon *et al.*, 2007).

It has recently been demonstrated that Arabidopsis and *B. napus* share well-conserved response mechanisms to CK treatment (Zuñiga-Mayo *et al.*, 2018). Strikingly, exogenous CK application causes a reduction in silique length in *B. napus*. However, these shorter siliques contain increased ovule numbers and upon manual pollination, the plants show an increase in seed yield of 18%. Intriguingly, increases in ovule and seed number have also been observed in the offspring of the treated plants, suggesting that the mechanism has an underlying epigenetic basis (Zuñiga-Mayo *et al.*, 2018).

An increase in CK level has also been reported to beneficially affect seed yield in transgenic *B. napus* lines expressing the CK biosynthetic enzyme isopentenyltransferase (*IPT*) under the Arabidopsis promoter of the *AtMYB32* gene. An increase in seed yield of up to 23% was obtained in the transgenic lines that were analysed (Kant *et al.*, 2015).

CK homeostasis is mediated by CYTOKININ OXIDASES/DEHYDROGENASES (CKXs) during pistil and silique development in Arabidopsis. Remarkably, the expression level of *CKX* genes in *B. napus* is associated with silique length, and RNA-sequencing and qRT-PCR analyses revealed a significantly different expression level of *BnCKX5-1*, *5-2*, *6-1*, and *7-1* in two distinct cultivated varieties with long versus short siliques (Liu *et al.*, 2018). These findings open up promising strategies with which to modulate silique length in *B. napus* by manipulating *CKX* gene expression.

In addition to phytohormones, genetic knowledge from Arabidopsis can be successfully applied to *B. napus* crop improvement. Mutations in the K-box of the Arabidopsis orthologue of *APETALA1* in *B. napus* caused a significant increase in the number of seeds per plant (Shah *et al.*, 2018). These generated alleles could conceivably be introduced into a rapeseed breeding programme in field trials.

Germplasm of *B. napus* revealed substantial natural variation with respect to seed number per pod. Current rapeseed cultivars produce on average 20 seeds per pod, which is far lower than the maximum observed among the germplasm resources (Yang *et al.*, 2017). Moreover, genetic improvement promises to deliver a massive improvement in seed yield (Yang *et al.*, 2017). The gold mine of knowledge obtained from the closely related species Arabidopsis will certainly be fundamentally important in the exploitation of the encouraging genetic variation potential. Furthermore, it has recently been demonstrated that CRISPR–Cas9 technology can be efficiently applied to precisely induce targeted mutation in rapeseed (Braatz *et al.*, 2017), making it a powerful tool for future genetic improvement. Similarly, existing knowledge could be used to improve other Brassicaceae species, or even non-phylogenetically related species such as soybean.
